# Biological Determinants of Metabolic Syndrome in Visceral and Subcutaneous Adipose Tissue from Severely Obese Women

**DOI:** 10.3390/ijms23042394

**Published:** 2022-02-21

**Authors:** Óscar Osorio-Conles, Arturo Vega-Beyhart, Ainitze Ibarzabal, José María Balibrea, Josep Vidal, Ana de Hollanda

**Affiliations:** 1 Centro de Investigación Biomédica en Red de Diabetes y Enfermedades Metabólicas Asociadas (CIBERDEM), Instituto de Salud Carlos III (ISCIII), 28029 Madrid, Spain; jovidal@clinic.cat; 2 Institut d’Investigacions Biomèdiques August Pi i Sunyer (IDIBAPS), 08036 Barcelona, Spain; beyhart@clinic.cat; 3 Gastrointestinal Surgery Department, Hospital Clínic de Barcelona, 08036 Barcelona, Spain; aibarza@clinic.cat (A.I.); balibrea@clinic.cat (J.M.B.); 4 Obesity Unit, Endocrinology and Nutrition Department, Hospital Clínic de Barcelona, 08036 Barcelona, Spain; 5 Centro de Investigación Biomédica en Red Fisiopatologia de la Obesidad y Nutrición (CIBEROBN), Instituto de Salud Carlos III (ISCIII), 28029 Madrid, Spain

**Keywords:** metabolic syndrome, obesity, subcutaneous adipose tissue, visceral adipose tissue

## Abstract

The metabolic syndrome (MetS) is a cluster of the most dangerous heart attack risk factors: diabetes or raised fasting plasma glucose, abdominal obesity, high cholesterol and high blood pressure. The goal of this study is to compare the state of the main features of obesity-associated white adipose tissue (WAT) dysfunction in 66 women with severe obesity without (MetS−) or with MetS (MetS+). Fat cell area, adipocyte size distribution and histological fibrosis were analysed in visceral (VAT) and abdominal subcutaneous WAT (SAT) in 33 age- and BMI-matched pairs of MetS− and MetS+ subjects. The mRNA expression of 93 genes implicated in obesity-associated WAT dysfunction was analysed by RT-qPCR in both fat depots. MetS+ females showed higher adipocyte hypertrophy in both fat depots and increased fibrosis and expression of macrophage and hypoxia markers in SAT. Transcriptional data suggest increased fatty acid oxidation in SAT and impaired thermogenesis and extracellular matrix remodelling in VAT from MetS+ subjects. A sPLS-DA model, including SAT expression of *PPARA* and *LEPR* genes identified MetS with an AUC = 0.87. Despite equal age, BMI and body composition, MetS+ females display morphological and transcriptional differences in both WAT depots, especially in SAT. These factors may contribute to the transition to MetS.

## 1. Introduction

The metabolic syndrome (MetS) is a cluster of conditions (raised fasting plasma glucose, abdominal obesity, high cholesterol and high blood pressure) that occur together and raises the risk of coronary heart disease, diabetes mellitus type 2, stroke and other atherosclerotic-associated diseases [1,2]. Although obesity with central fat distribution is considered key to the clustering of the MetS components, a significant proportion of persons living with obesity (PLWO) do not present the MetS [3].

White adipose tissue (WAT) dysfunction has emerged as a major determinant of obesity-related metabolic complications, and the clinical presentation of MetS might be to some extent based on disrupted physiology of the WAT [4,5,6,7]. WAT dysfunction has been associated with an insufficient angiogenic potential [8], unresolved inflammation [8,9], an altered adipokine secretome [10,11] and inappropriate extracellular matrix (ECM) remodelling [8]. The causal order in this context is not completely known, but hypertrophic adipocytes seem more prone to this scenario as they reach the diffusional limit of oxygen, resulting in persistent hypoxia and ultimately leading to unhealthy WAT tissue expansion [8]. Visceral WAT (VAT) depot, in opposition to subcutaneous (SAT), is more cellular; vascular; innervated; more sensitive to adrenergic stimulation; contains a larger number of inflammatory and immune cells and their adipocytes are more metabolically active, hypertrophic and insulin-resistant, carrying thus a greater prediction of mortality than SAT [12]. Consequently, insulin resistance has been associated with visceral adiposity in PLWO [13]. The current knowledge about adipose tissue implications on the pathophysiology of MetS has been remarkably summarized [14], and despite several adipose-derived miRNAs [15] and hormones [16,17,18,19,20], were found associated with MetS, and the role of WAT function on MetS is still poorly understood.

Although comparisons of PLWO with the MetS (MetS+) versus without the MetS (MetS−) patients have been widely reported at a systemic level or in relation to body composition [21,22,23,24], reports evaluating histological and transcriptomic differences in WAT between these populations are scarce. Moreover, most of these studies groups were not matched for key confounders like age, weight, total body mass or central fat distribution. Thus, Bremer et al. found increased macrophage recruitment [25], fibrosis and angiogenesis [26] in gluteal SAT of MetS patients, although the groups were not BMI-matched, and most controls were not obese. Similarly, Viguerie et al. found a MetS signature in SAT for 22 genes common to men and women with obesity [27], although subjects with the MetS had higher age, weight, BMI and adiposity. Therefore, the goal of our study was to compare the histology and transcriptomic profile of obesity-associated WAT dysfunction in age- and BMI-matched pairs of females with severe obesity with (MetS+) or without (MetS−) the MetS in order to test the hypothesis that a WAT signature is associated to the presence of MetS.

## 2. Results

### 2.1. Anthropometric and Clinical Data

The clinical characteristics of the 66 female participants are shown in Table 1. As expected, all MetS parameters were significantly different between groups, and the proportion of subjects with T2D (*p* = 0.001) and HTN (*p* < 0.0001) was larger in the MetS+ group. In addition, the percentage of glycosylated hemoglobin (HbA1c, *p* = 0.037) was higher in subjects in the MetS+ group. Total and low-density lipoprotein- (LDL) cholesterol were similar between groups.

The presence of non-alcoholic fatty liver disease (NAFLD, *p* = 0.028), serum gamma-glutamyl transferase (GGT) levels (*p* = 0.009), fatty liver index (FLI, *p* = 0.018) and the triglyceride glucose index, a surrogate marker of insulin resistance (TyG, *p* < 0.001), were higher in MetS+ individuals. However, composite indices for liver fibrosis (FIB-4 and APRI) were comparable between study groups. Finally, high-sensitivity C-reactive protein (hs-CRP) levels did not differ between groups.

Of note, body composition variables assessed by DXA scan, such as total body, android and gynoid fat and estimated VAT masses, were comparable between the two study groups after matching (Table 2).

### 2.2. Fat Cell Size Distribution and Fibrosis

**Figure 1:**
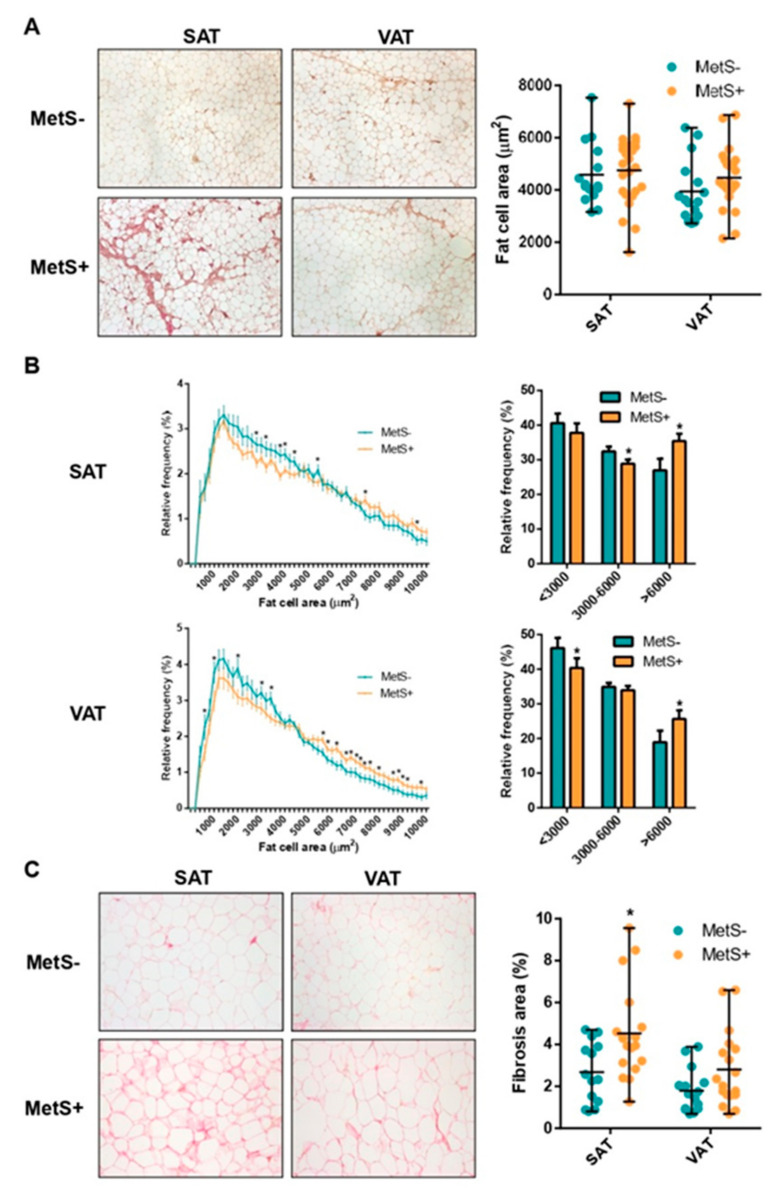
Fat cell size distribution and fibrosis. Comparison of adipocyte cell surface area and representative images (**A**) of SAT and VAT samples from MetS− and MetS+ individuals. Frequency distribution analysis of fat cell areas divided by size into bin intervals of 200 µm^2^ and into three representative sizes (**B**). Data are presented as the average ± SD frequencies of cells within each bin and compared by the Holm–Sidak *t*-test for multiple comparisons or by Welch’s *t*-test among size intervals. Comparison of histological pericellular fibrosis and representative images (**C**). Data are presented as the ratio of fibrous tissue area stained with picrosirius red/total tissue surface. SAT, subcutaneous adipose tissue; VAT, visceral adipose tissue; MetS−, severely obese without MetS; MetS+, severely obese with MetS. * = *p* < 0.05.

The mean adipocyte area was similar in both SAT and VAT samples from MetS− and MetS+ female individuals (Table 2 and Figure 1A). Nevertheless, when adipocyte areas were divided by size into bin intervals of 200 µm^2^, frequency distribution analysis showed a significant decreased proportion of medium-sized and an increased percentage of larger adipocytes at various cell sizes in SAT from MetS+ subjects. Similarly, VAT from MetS+ individuals had a lesser proportion of smaller and a greater proportion of larger adipocytes at various size intervals (Figure 1B). Sirius red staining on formalin-fixed sections showed that fibrosis around adipocytes (i.e., pericellular fibrosis) was 1.7-fold higher in SAT samples from MetS+ individuals (*p* = 0.008), while a nonsignificant trend (*p* = 0.052) was found in VAT (Table 2 and Figure 1C).

### 2.3. Differential Gene Expression Analysis

Descriptive statistics of the gene expression analysis of the total of 93 selected genes implicated in WAT dysfunction evaluated in both fat depots and grouped by MetS status are shown in Supplementary Tables S1–S3. Sixteen genes (17%) in SAT and 16 genes (17%) in VAT were differentially expressed in MetS+ versus MetS− subjects (Table 3). Amongst the studied genes, only *FASN*, *CPT1A*, *TGFB1* and *F13A1* were differently expressed in both fat depots, all of them in the same direction. All the differently expressed SAT genes were upregulated in the MetS+ patients, except for *FASN*, which was downregulated in both adipose compartments, and *LEPR*. In VAT, 11 genes were downregulated in MetS+ patients, while *MSR1*, *GLUT1*, *CPT1A*, *TGFB1* and *F13A1* were upmodulated. *TGFB1* and *F13A1* were upregulated in both SAT and VAT depots. In MetS− individuals, a total of 49 genes (52%) were differentially expressed in VAT versus SAT (27 up- and 22 downmodulated). In MetS+ subjects, a distinct expression profile was found for 44 genes between fat depots (47%), of which 14 were upregulated.

### 2.4. Expression of Inflammation Markers, Adipokines and Genes Implicated in WAT Expansion

The mRNA levels of M1-type (*CD80*), M2-type (*MRC1*/*CD206*) and total macrophage (*CD68*) markers were increased by 3-, 1.35- and 1.47-fold in SAT from MetS+ versus MetS− subjects, while, in VAT, only the M2-type marker *MSR1*/*CD204* showed a slight 1.28-fold upregulation (Table 3). In addition, the subcutaneous expression of hypoxia-inducible factor 1-alpha (*HIF1A*) and adiponectin (*ADIPOQ*) was upregulated in MetS+ subjects by 1.52- and 1.39-fold, respectively, while leptin receptor (*LEPR*) was downregulated by 0.62-fold. No differences were observed in the gene expression of senescence and angiogenic genes in relation to the presence of MetS (Supplementary Table S1). The adipogenic marker peroxisome proliferator-activated receptor alpha (*PPARA*) was found 1.25-fold upregulated in SAT, while fatty acid-binding protein 4 (*FABP4*) was decreased by 0.53-fold in VAT from MetS+ patients.

### 2.5. Expression of Genes Implicated in Glucose and Lipid Metabolism and Fatty Acid Oxidation (FAO)

Monoacylglycerol acyltransferase 1 (*MOGAT1*) was increased by 1.5-fold in MetS+ subjects’ SAT, while glucose transporter 1 (*GLUT1*) mRNA levels were higher (1.33-fold) in VAT (Table 3 and Supplementary Table S2). Conversely, fatty acid synthase (*FASN*) was downregulated in both SAT (0.53-fold) and VAT (0.64-fold) depots from MetS+individuals.

In MetS+ patients, beta-adrenergic receptor 1 (*ADRB1*) mRNA levels were increased (1.73-fold) in SAT, while *ADRB3* levels were decreased (0.33-fold) in VAT. In SAT, the beiging marker PR/SET domain 16 (*PRDM16*) was two-fold upregulated. Meanwhile, in VAT, the thermoregulatory gene cell death-inducing DFFA-like effector A (*CIDEA*) and the functional marker of both brown and beige adipocytes uncoupling protein 1/thermogenin (*UCP1*), were downregulated by 0.64- and 0.4-fold, respectively. For its part, carnitine palmitoyltransferase 1A (*CPT1A*), a key enzyme in fatty acid β-oxidation, was upregulated in both fat depots from MetS+ group (1.37- and 1.41-fold in SAT and VAT, respectively).

### 2.6. Expression of ECM Components and Modifiers

A number of transcripts implicated in ECM composition and remodelling were screened and several genes were found associated to the MetS status. Thus, the production of the pro-fibrotic cytokine TGF-β1 was slightly upregulated (1.3-fold) in both SAT and VAT of MetS+ individuals (Table 3 and Supplementary Table S3). Similarly, the mRNA levels of coagulation factor XIII A chain (*F13A1*), another proinflammatory factor implicated in tissue remodelling pathways, were 1.71- and 1.61-fold higher in SAT and VAT, respectively. Besides, two lysyl oxidase homologs, lysyl oxidase-like 2 (*LOXL2*) and 4 (*LOXL4*), responsible for elastin cross-linking within the ECM were found 1.4-fold upregulated in SAT from MetS+subjects. Finally, one ECM component and five ECM modifiers were downregulated in VAT from MetS+individuals. This was the case for collagen type V alpha 1 chain (COL5A1, 0.68-fold), matrix metalloproteinases 2 (*MMP2*, 0.51-fold) and 13 (*MMP13*, 0.1-fold), the metalloproteinase inhibitor 1 (*TIMP1*, 0.45-fold) and the hyaluronidases 1 (*HYAL1*, 0.29-fold) and 2 (*HYAL2*, 0.28-fold). Other transcripts differed among fat depots but not in relation to the MetS status (Supplementary Table S3).

### 2.7. Principal Component Analysis (PCA) to Identify MetS Subjects

**Figure 2:**
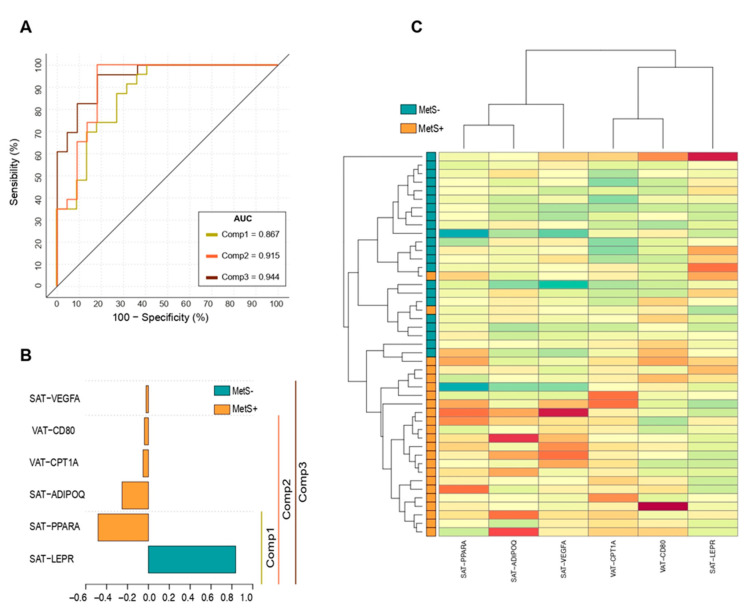
Subcutaneous and visceral fat gene expression signature model for MetS. (**A**) sPLSDA receiver operating characteristic (ROC) analysis of the gene expression signature components. Lines correspond to the accuracy classification performance of each component, including the previous. (**B**) sPLSDA loading plot. Bar length corresponds to each gene loading weight (importance) into the model. Bar color indicates the group in which the mean gene expression is higher. (**C**) Gene expression hierarchical dendrogram heatmap of the transcriptome signature. Euclidean distance metric with Ward’s group linkage algorithm was performed to cluster groups. MetS−, severely obese without MetS; MetS+, severely obese with MetS.

Unsupervised PCA including gene expression data from all 93 SAT and VAT genes did not clearly separate MetS+ and MetS− groups (Supplementary Figure S1). A sPLS-DA model was then tunned with three optimal components to deconstruct the transcriptome data based on the lowest balanced errors. The number of genes per component was selected assessing the mean squared error of prediction of the class. A 10-fold cross-validated transcriptome signature model (R2Y = 0.91; Q2 = 0.45, *p* ≤ 0.001) composed by the expression of six genes resulted in a correct classification of patients with an area under the ROC curve of 0.94 (*p* < 0.001, Figure 2A). The expression of leptin receptor in SAT (SAT-*LEPR*) had the highest contribution to the classification model, being the only gene of the model upregulated in MetS− patients (Figure 2B). Five genes in the model were therefore upregulated in the MetS+ group, including SAT expression of *PPARA*, *ADIPOQ* and *VEGFA* and VAT expression of *CD80* and *CPT1A*. The signature heatmap showed a clear hierarchical division between genes associated with the presence of MetS (Figure 2C). Multinomial logistic regressions were then performed to assess the expression association of those genes included in the signature model on the likelihood that patients have MetS. SAT-*LEPR* and SAT-*PPARA* explained 64.4% of the variance of MetS (*p* = 0.000) being the only genes independently associated with this condition (Table 4). Females with higher expressions of SAT-*LEPR* were less likely to present MetS (0.12 (0.04–0.43)), while those with increased SAT-*PPARA* were 6.01 (1.27–12.95) more likely to have the condition. Age and listed medications were not associated with the gene expression models (*p* > 0.05).

MetS discrimination models combining histology markers (SAT and VAT fibrosis and mean fat cell area) and gene-expression were then tested by polynomial-logistic regression with forward conditional selection method. VAT-fibrosis showed an independent association to MetS when included in a binary logistic model with SAT-*LEPR* expression (β = 1.35 [1.18–12.9], *p* = 0.002). However, discrimination power was lower (R^2^ = 0.52, correct classification = 80.8%) than models, including only gene-expression data (Table 4). SAT and VAT adipocyte area as well as SAT-fibrosis were excluded due to non-prediction improvement of the fitted models.

## 3. Discussion

This study aimed to identify obesity-associated WAT alterations in relation to the presence of the MetS, diminishing the effect of confounders by selecting a BMI- and age-matched cohort. A population composed exclusively of women was considered to reduce variability in terms of body fat distribution and to avoid known gender-based disparities in metabolic inflammation, adipocyte function and dynamics [28,29,30]. MetS+ patients showed alterations in plasma levels of triglycerides, HDL cholesterol, glucose and hepatic parameters of liver function in relation to MetS− subjects, while circulating levels of coagulation and inflammatory markers did not differ between groups. This is in line with the major role that atherogenic dyslipidemia and insulin resistance play in the MetS [31].

Key findings of our study include the presence of a greater proportion of hypertrophic adipocytes in both fat depots and increased SAT pericellular fibrosis in patients with the MetS despite comparable visceral and subcutaneous adiposity. MetS+ subjects displayed transcriptomic differences in WAT, especially in genes belonging to pathways related to inflammation, FAO, thermogenesis and ECM remodelling. Moreover, lower SAT-*LEPR* and higher SAT-*PPARA* expression was independently associated to the likelihood of presenting the MetS, highlighting the implication of the lipid metabolism in the pathophysiology of the disease.

WAT expands by either adipocyte enlargement (hypertrophy) or preadipocyte recruitment (hyperplasia). Adipocyte hypertrophy and aberrant ECM deposition have been consistently characterized as important features of the obesity-associated WAT dysfunction [32,33] predisposed to the development of the MetS [34,35]. In our study, despite similar BMI and body composition, MetS+ individuals showed a greater proportion of hypertrophic adipocytes in both fat depots. In addition, we found increased fibrosis assessed by Sirius red staining in abdominal SAT from MetS+ subjects, in agreement with previous findings in gluteal SAT from MetS patients [25,26].

A number of genes were differentially expressed in SAT and VAT between study groups, and within each group, a number of genes showed different expression levels among the two fat depots. Nevertheless, while, in MetS−, most of such genes were upregulated in VAT, the expression of visceral genes in MetS+ subjects were more frequently downmodulated. Overall, a similar relative expression profile was found among depots in both study groups, except for those genes implicated in inflammation, WAT expansion and senescence.

It has been proposed that hypertrophic adipocytes are prone to reach the diffusional limit of oxygen, resulting in persistent hypoxia and ultimately leading to unhealthy WAT tissue expansion [8]. In this sense, we found in SAT from MetS+ an upregulation of the master regulator of O2 homeostasis *HIF1A*. Hypoxia, for its turn, is a major trigger for WAT inflammation present in MetS [36]. We observed an upregulation of markers of M1 (CD80), M2 (MRC1) and total macrophages (CD68) in SAT from MetS+ group. Interestingly, we observed an upmodulation of two different M2-macrophage markers, *MRC1* and *MSR1* in SAT and VAT, respectively, suggesting the recruitment of different M2 subpopulations between depots in relation to the MetS. Of note, visceral levels of M1 (CD80+) and pan-macrophage markers (CD68+) were comparable between groups. This counterintuitive finding might be partly explained by comparable expression of *HIF1A* and angiogenesis genes in VAT from our BMI-matched groups.

Although no differences were found in the expression of lipolytic enzymes in our study, we observed an upregulation in SAT from MetS+ subjects of genes that modulate lipolysis, FAO or thermogenesis (*ADRB1* [37,38], *PPARA* [39,40] and *PDRM16* [41]. On the contrary, several genes related to these pathways such as *ADRB3* [38], *UCP1* [42] and *CIDEA* [43], were downmodulated in MetS+ patients’ VAT. Interestingly, *CPT1A* the rate-limiting enzyme in FAO was found upregulated in both fat depots, and its visceral expression was the fourth gene among the MetS signature contributors.

Adipose tissue ECM composition and remodelling is complex and context-dependent [44]. Although higher histological fibrosis was only found in SAT, we report an upregulation of the pro-fibrotic cytokine TGF-β1 and the tissue transglutaminase enzyme F13A1 in both fat depots from MetS+ group. Furthermore, a number of genes implicated in ECM assembly and remodelling was found dysregulated in females with the MetS, especially within the VAT depot. Adipose tissue MMPs are synthesized by different cells types -endothelial cells, fibroblasts, adipocytes, immune cells- and play important roles in physiological processes such as cell proliferation and angiogenesis, but they have also been involved in pathological processes including adipogenesis and fibrosis [45]. HYALs catalyse the hydrolysis of hyaluronan, a nonsulfated linear glycosaminoglycan polymer, abundantly present in WAT-ECM and important for its maturation [46]. Nevertheless, the potential causal role of HYALs on hyaluronan accumulation and fibrosis, and the implication of hyaluronan turnover on metabolic disease sequelae remain to be elucidated [46].

Overall, in our study both HYALs and MMPs were among the most downmodulated genes in VAT from MetS+ individuals and this was associated with a lower abundance of smaller (<3000 µm^2^) and higher abundance of larger adipocytes (>6000 µm^2^). Such results could be interpreted as a diminished ability for visceral ECM remodelling in obese females with the MetS. In this sense, epididymal adipose gene signatures related to disease pathogenesis have been recently identified in a mouse model of the MetS [47] where inflammation and ECM-related pathways were found overrepresented. However, divergences among depots and differences in WAT-ECM composition between mouse models and humans should be taken into account.

Our model including SAT expression of *LEPR* (decreased) and *PPARA* (increased) correctly classified 85% of MetS cases. SAT-*LEPR* mRNA levels thus corresponded to the highest loading weight in our model. It is well known that obesity is associated with hyperleptinemia and leptin resistance due to changes in leptin transport across the blood-brain barrier, as well as in LEPRs or their isoforms [48]. WAT expresses both the short and long isoforms of leptin receptors [49,50], not only in adipocytes but also in endothelial and immune cells [51] and, peripheral in parallel to central leptin resistance can occur. Thus, mRNA levels of the long isoform have been found 90% decreased in SAT from morbidly obese versus lean women [52]. While leptin suppresses adipocyte proliferation through central LEPRs [53], local action on adipocyte LEPRs seems to induce proinflammatory cytokine profile and promote adipogenesis [54], as well as lipolysis, browning and apoptosis [55]. Conversely in our study these pathways did not seem to be downmodulated in SAT from MetS+ group despite lower SAT-LEPR expression, neither higher local leptin resistance was countered by higher leptin production.

On the other hand, PPARA is a transcriptional modulator of fatty acid transport, peroxisomal and mitochondrial FAO pathways [56] and, thus, through its pro-oxidative anti-lipotoxic effects, their ligands are successfully used to treat primary and secondary forms of hypertriglyceridemia, particularly associated with MetS [57]. Of note, gene expression of *PPARA* in both fat pods was not modulated by fibrate consumption in our cohort, and despite similar levels in VAT, SAT-*PPARA* was found upregulated in MetS+ females being a main predictor of MetS.

Additional factors involved in our model include SAT expression of *ADIPOQ* and *VEGFA*, and VAT expression of *CPT1A* and *CD80*. While VAT-*ADIPOQ* mRNA levels were comparable in both groups, we found increased SAT-*ADIPOQ* expression in MetS+ females, being the third gene in the signature contributors. Adiponectin is the most abundant protein secreted by WAT, has insulin-sensitizing, anti-atherogenic and anti-inflammatory effects [58], and its circulating levels are positively associated with insulin sensitivity and decrease with obesity [59,60], being thus proposed as a therapeutic target [61,62]. ADIPOQ plasma circulating levels have been consistently found decreased in MetS patients [16,17,18], but again, age, BMI, waist circumference, percent body fat, SAT and VAT fat area were repeatedly higher in MetS groups. In addition, dissociations of SAT-ADIPOQ mRNA with its plasma circulating levels have been previously reported [63,64] and comparable subcutaneous expression [65] and secretion [18] were found between MetS and control lean patients. In this sense, it has been suggested that the main contributor of circulating ADIPOQ is VAT depot, as the secretion rate from the omental region is significantly higher than that from SAT [66] Of note, higher *ADIPOQ* expression in our study was related to increased mRNA levels of FAO-related genes and anti-inflammatory macrophage markers, as previously reported [67,68,69,70].

Although no differences were previously found in WAT CPT1A mRNA expression in relation to BMI or diabetic status [71], an inverse association between methylation at intronic loci of CPT1A and MetS as well as individual MetS components have been identified [72]. Overall, our results suggest a promotion of FAO activity in both fat depots, along with an impaired thermogenic capacity in VAT from MetS+ individuals. Despite VAT-*CD80* and SAT-*VEGFA* expression were not significantly different between groups, both genes contributed at a lesser extent to the MetS transcriptomic signature. Increased SAT expression of vascular endothelial growth factor-A (VEGFA) showed the least discriminatory power in our model. VEGFA controls WAT function and systemic energy metabolism not only by modulating adipose vasculature [73] but also promoting WAT browning [73,74,75]. VEGFA circulating levels are raised in obesity [76] and secretion from omental VAT seems to be the main contributor [77]. Nevertheless, a recent metanalysis suggested an overexpression of VEGFA in subjects with MetS, hypertension, hypertriglyceridemia and hyperglycaemia, but not in relation to obesity or high LDL [78].

Viguerie et al. previously found a MetS signature for 22 genes in SAT from obese females which included a reduced expression of FASN [27]. FASN catalyses the last step in the fatty acid biosynthetic pathway and is believed to be a determinant of the maximal capacity of de novo lipogenesis [79]. Although we found a significant decrease in FASN expression in both fat depots from MetS+ subjects, its expression was not among the genes that best identified the presence of MetS. Matching for age and BMI in our study could affect its relevance.

Finally, we acknowledge our study is not without limitations. First, our study population was composed exclusively by women. As mentioned above, we chose to include females only in order to reduce variability in terms of body fat distribution and gender-based disparities in WAT function. This should prevent drawing conclusions on a male population. Second, women with exclusively severe obesity were considered. This may limit the scope of our results on overweight and mildly obese women populations. Third, it cannot be excluded a potential impact of ethnicity on SAT and VAT distribution and function [80]. Ultimately, the limited number of subjects included in our study could have hampered our ability to find significant components in our signature model.

## 4. Materials and Methods

### 4.1. Study Population

White adipose tissue samples were collected from 128 female subjects with severe obesity undergoing bariatric surgery at the Obesity Unit of the Hospital Clínic of Barcelona between April 2018 and September 2020. Of them 50 did not fulfil the criteria for the diagnosis of the MetS (MetS−, 39%) and 78 presented ≥3 components of the MetS (MetS+, 61%) according to the IDF definition. The study’s exclusion criteria were previous bariatric surgery, a history of malignancy, chronic inflammatory diseases, active infectious diseases, drug abuse or daily alcohol consumption >20 grams. One-to-one propensity score matching (PSM) methodology (nearest neighbour) with a maximum allowed distance of Δ0.001 was used to obtain 1-1 age and BMI matched pairs of female subjects with (MetS+) and without MetS (MetS−). Thirty-three matched pairs were identified. Ethics committee approval conforming to the Declaration of Helsinki for sample collection was obtained from the Clinical Research Ethics Committee (CEIC) of Hospital Clinic de Barcelona (R120615-084, 13 October 2016). All participants provided written informed consent.

### 4.2. Clinical and Anthropometric Data

The patients’ anthropometric measurements were collected following standardized procedures and haematological and biochemical parameters were determined at the Core Laboratory of the Biomedical Diagnostic Center using an Advia 2400 analyser (BayerDiagnostics, Tarrytown, NY, USA), as previously reported [81]. Anthropometric and clinical data are summarized in Table 1.

### 4.3. Determination of Metabolic Health Status

Presence of MetS was ascertained as part of the pre-bariatric surgery evaluation and following the MetS criteria of the IDF [2]. Thus, MetS was present if the female patient had BMI ≥ 30 kg/m^2^ plus two or more of the following four factors: (1) raised concentration of triglycerides: ≥150 mg/dL (1.7 mmol/L) or specific treatment for this lipid abnormality; (2) reduced concentration of HDL cholesterol: <50 mg/dL (1.29 mmol/L) or specific treatment for this lipid abnormality; (3) raised blood pressure: systolic blood pressure ≥ 130 mmHg or diastolic blood pressure ≥ 85 mmHg or treatment of previously diagnosed hypertension; and (4) raised fasting plasma glucose concentration ≥ 100 mg/dL (5.6 mmol/L) or previously diagnosed type 2 diabetes.

### 4.4. Body Composition

Total body fat and lean mass were measured by dual-energy X-ray absorptiometry (DXA) scan using a GE Lunar iDXA with the software enCORE provided by the manufacturer (GE Healthcare, Madison, WI, USA). The software was also used to calculate estimated visceral fat (eVAT) in the android region from the following formula: total adipose fat mass in the android region = eVAT + estimated subcutaneous adipose tissue in the android region.

### 4.5. White Adipose Tissue Biopsies

Paired abdominal SAT and omental VAT samples were obtained at the time of surgery as previously reported [82]. Samples were collected in DMEM and rinsed in PBS. A portion was immediately frozen before RNA analysis. Other portion was fixed overnight at 4 °C in 4% paraformaldehyde and processed for standard paraffin embedding. Starting at the tissue apex 3 × 3-μm-thick sections were made at a minimum of 100 μm intervals across the sample tissue.

### 4.6. Morphometry and Histopathology

Hematoxylin and eosin (H&E) staining was used to assess adipocyte morphology. Digital images were captured under an Olympus ×600 microscope (Tokyo, Japan) at 4× magnification. Adipocyte size was measured within micrographs of at least 3000 cells per sample from randomly selected fields using Adipocytes Tools, an ImageJ macro-based algorithm for ImageJ software (National Institutes of Health, Bethesda, MD, USA; http://imagej.nih.gov/ij/). Adipocyte average area and size distribution was calculated and frequency distribution analysis into bin intervals of 200 µm^2^ was performed.

Sirius red staining was used for quantification of pericellular fibrosis (i.e., extracellular matrix accumulation around the cells) [83]. Automated analysis of the captured images at 10× magnification has been carried out using MRI Fibrosis Tool, an ImageJ macro-based algorithm, and expressed as a percentage of red staining (fibrosis)/tissue surface ratio.

### 4.7. RNA Extraction and Real Time PCR

Total RNA was isolated using RNeasy Lipid Tissue Mini Kit (Qiagen, Hilden, Ger-many). Concentration and purity were measured using a NanoDrop 1000 spectrophotometer (Thermo Scientific, Waltham, MA, USA). Equal amounts of RNA from SAT and VAT (2 μg) were reverse-transcribed using the Superscript III RT kit and random hexamer primers (Invitrogen, Carlsbad, CA, USA). Reverse transcription reaction was carried out for 90 min at 50 °C and an additional 10 min at 55 °C. An expression analysis of 93 genes involved in WAT dysfunction and related to inflammation, adipogenesis, autophagy, fatty acid metabolism and oxidation, adipocyte britening, glucose metabolism and adipokines was performed in both fat depots. Real-time quantitative PCR (qPCR) was performed with a 7900HT Fast Real-Time PCR System (Applied Biosystems, Foster City, CA, USA) using GoTaq^®^ qPCR Master Mix (Promega Biotech Ibérica, Madrid, Spain). Expression relative to the housekeeping gene RPL6 was calculated using the delta Ct (DCt) method. Gene expression is presented as the 2^(-DCt) values. The list of primers used in this study is provided in Supplementary Table S4.

### 4.8. Circulating Levels

Serum levels of GM-CSF, IL-1β, IL-6 and TNF-α, were measured in plasma samples by using magnetic bead Milliplex MAP™ custom panels (EMD Millipore, Burlington, MA, USA) following the supplier’s instructions. Data from the reactions was acquired using the Luminex 100™ System (Luminex, Austin, TX, USA) and analysed as fluorescence intensity. Thereafter, data was processed and analysed with the Milliplex Analyst™ v.5.1.0.0 standard, 2012 (Merck Millipore KGaA, Darmstadt, Germany) and presented as target concentrations.

### 4.9. Statistics

Continuous data with normal and non-normal distribution is expressed with arithmetic means and standard deviations (SD), or medians and interquartile ranges (IQR), respectively. Categorical variables are expressed with frequencies and proportions. No data transformation was performed on gene expression when performing univariate analysis and normality assumption was tested with Shapiro–Wilk test. Linearity, and absence of multicollinearity were also checked. Mann–Whitney U test, Welch’s t-test or Student’s t-test were used when adequate to assess the magnitude of the difference between groups. Mean gene expression fold changes were calculated with the MetS−group as the reference. To reduce confounding, the degree of association between age, sex and ongoing medical treatments associated with MetS (metformin, insulin, GLP1-ra, DPP4i, SGLT2i, statins and cholesterol absorption inhibitors) with the expression of each of the genes analysed were assessed and adjusted in multivariate models if significance was found.

Component analysis and multivariate dimensionality reduction methods were used to find a WAT-transcriptomic signature for MetS. Missing gene expression values (*n* = 863, 19.7%) were imputed with the k-nearest neighbour algorithm and standardized to zero means and unit variances. To examine the intrinsic dimension and general structure of the transcriptome across groups, principal component analysis (PCA) using singular value decomposition was implemented. Leave-one-out cross-validation of a sparse partial least square discriminant analysis (PLS-DA) was then used to determine the optimal number of components and genes to be included in the signature model. The number of components was chosen based on the estimation of the lowest balanced error rate (BER) and the genes based on the lowest prediction error for each subset of genes on each component. R2Y (the sum of squares) and Q2Y (the predictive performance) values were assessed to ensure the absence of overfitting of the final model. Area Under the Curve (AUC) values were calculated from the predicted scores in the LOO cross-validation process minimizing the risk of overfitting. Heatmap was created employing multivariate Euclidean distance metric with complete linkage method and presented with associated dendrograms. Finally, Spearman correlations followed by polynomial regressions with stepwise method were then performed to identify genes independently associated with the presence of MetS among those included in the model.

All the comparisons stated as different in the present manuscript have statistical significance with a two-tailed *p*-value < 0.05. GraphPad PRISM 6.0, Statistical Package for Social Sciences software (SPSS, version 25.0, Chicago, IL, USA) and R (The R Project for Statistical Computing) software environment [84] were used to perform the analyses.

## 5. Conclusions

Through matching patients by age and BMI we could assess the obesity-associated alterations of the adipose tissue present in MetS limiting possible confounders. Overall, both fat depots from MetS+ subjects showed higher numbers of hypertrophic adipocytes and traits of increased fat oxidation. SAT also displayed a higher degree of fibrosis and expression of macrophage markers. This could be interpreted as an adaptive response to limit adipocyte hypertrophy and to compensate the metabolic dysfunction. On the contrary, disrupted VAT-ECM remodelling and thermogenesis may be contributing factors to the development of MetS. A model combining decreased SAT-mRNA levels of *LEPR* together with the upregulation of *PPARA* levels showed a high performance in discriminating the presence of MetS in our female cohort. Interestingly, although VAT is generally considered a major correlate of MetS. most genes included in our signature model were expressed in SAT. These results emphasize the contribution of SAT biology in metabolic disorders and suggest potential early risk signs of MetS in SAT from females with severe obesity.

## Figures and Tables

**Table 1:** Clinical characteristics of the study patients.

	MetS− (*n* = 33)	MetS+ (*n* = 33)	*p*-Value
Age (years) ^a^	47.97 ± 8.81	49.24 ± 10.65	0.599
BMI (kg/m^2^) ^ab^	43.60 ± 5.12	44.12 ± 4.06	0.651
Waist (cm) ^b^	123.29 ± 13.45	122.2 ± 10.51	0.785
Hip (cm)	137.64 ± 9.67	134.48 ± 8.73	0.312
Waist-to-hip ratio	0.90 ± 0.07	0.91 ± 0.06	0.516
HTN ^b^	2 (6.06%)	18 (54.54%)	<0.0001 ^c^
TG (mg/dL) ^b^	109.34 ± 38.26	147.15 ± 45.7	0.001
Total cholesterol (mg/dL)	193.82 ± 24.44	202.55 ± 36.2	0.337
HDL (mg/dL) ^b^	53.85 ± 9.01	47.15 ± 8.9	0.003
LDL (mg/dL)	118 ± 22.18	123.13 ± 26.96	0.408
FPG (mg/dL) ^b^	96.36 ± 22.68	110.12 ± 22.49	<0.0001
T2D	1 (3.03%)	12 (36.36%)	0.001
HbA1c (%)	5.69 ± 0.78	6.04 ± 0.87	0.037
AST (IU/L)	20.84 ± 7.47	23.3 ± 6.83	0.103
ALT (IU/L)	25.48 ± 14.62	29.42 ± 18.42	0.185
GGT (IU/L)	24.81 ± 17.24	37.15 ± 25.5	0.009
AST:ALT ratio	0.94 ± 0.35	0.95 ± 0.42	0.914
Platelets (×10^9^/L)	273.88 ± 54.16	305.61 ± 75.74	0.268
hs-CRP (mg/dL)	0.82 ± 0.69	1.36 ± 1.67	0.134
GM-CSF (pg/mL)	11.92 (9.52–19.59)	19.44 (7.28–29.38)	0.403
IL-1ß (pg/mL)	0.85 ± 0.55	0.90 ± 0.48	0.849
IL-6 (pg/mL)	0.89 ± 0.53	1.14 ± 0.95	0.48
TNFα (pg/mL)	2.90 ± 0.91	3.64 ± 1.85	0.526
NAFLD	19 (57.58%)	28 (87.5%)	0.028 ^c^
FIB-4 Score	0.81 ± 0.38	0.79 ± 0.33	0.769
APRI Score	0.20 ± 0.11	0.20 ± 0.07	0.312
HSI Index	54.73 ± 7.02	56.6 ± 4.42	0.09
TyG Index	4.59 ± 0.21	4.81 ± 0.19	<0.0001
FLI Index	94.57 ± 5.24	97.51 ± 1.87	0.018

Data are presented as the mean ± SD, median (IQR) or number (%). MetS−, severely obese without MetS; MetS+, severely obese with MetS; BMI, body mass index; HTN, hypertension; TG, serum triglyceride level; HDL, serum high-density lipoprotein cholesterol level; LDL, serum low-density lipoprotein cholesterol level; FPG, fasting plasma glucose; T2D, type 2 diabetes; HbA1c, glycosylated haemoglobin; AST, serum aspartate aminotransferase level; ALT, serum alanine aminotransferase level; GGT, gamma-glutamyl transferase; hs-CRP, high-sensitivity C-reactive protein; GM-CSF, granulocyte-macrophage colony-stimulating factor; IL, interleukin; TNFα, tumor necrosis factor alpha; NAFLD, nonalcoholic fatty liver disease; FIB-4, index for liver fibrosis; APRI, AST to platelet ratio index; HSI, hepatic steatosis index; TyG, triglyceride glucose index; FLI, fatty liver index. ^a^ Categorical criteria for PSM. ^b^ Categorial citeria for MetS. ^c^ Fisher’s exact test.

**Table 2:** Body composition characteristics and adipose tissue histological variables.

	MetS−	MetS+	*p*-Value
Total mass (kg)	105.5 (97.65–119.4)	108.8 (105.6–122.9)	0.394
Lean mass (g)	48,399 ± 6280	49,637 ± 5737	0.591
Fat-free mass (g)	50,829 ± 6444	52,037 ± 5968	0.611
Fat mass (g)	56,637 (50,181–63,045)	58,213 (51,129–66,602)	0.546
Android fat mass (%)	60.5 (57–63.55)	61.3 (58.2–65.6)	0.446
Gynoid fat mass (%)	56 (52.6–59.4)	55.6 (51.8–58)	0.675
Total fat mass (%)	54.9 (50.2–57.05)	54.7 (51.5–57.7)	0.742
eVAT (g)	1904 ± 699	2276 ± 696.1	0.179
eVAT (cm^3^)	2018 ± 741	2412 ± 738.1	0.179
SAT fat cell area (µm^2^)	4585 ± 1195	4747 ± 1290	0.689
VAT fat cell area (µm^2^)	3941 ± 1182	4470 ± 1130	0.168
SAT:VAT fat cell area ratio	1.25 ± 0.29	1.06 ± 0.19	0.041
SAT Total fibrosis (% area)	0.94 ± 0.39	1.10 ± 0.39	0.277
VAT Total fibrosis (% area)	0.74 ± 0.35	0.92 ± 0.44	0.259
SAT Pericellular fibrosis (% area)	2.68 ± 1.45	4.52 ± 2.21	0.029
VAT Pericellular fibrosis (% area)	1.80 ± 1.06	2.80 ± 1.91	0.097
SAT:VAT fibrosis ratio	1.44 ± 1.31	1.70 ± 0.92	0.222

Data are presented as the mean ± SD or median (IQR). MetS−, severely obese without MetS; MetS+, severely obese with MetS; eVAT, estimated visceral adipose tissue; SAT, subcutaneous adipose tissue, VAT, visceral adipose tissue.

**Table 3:** Differential gene expression analysis.

	MetS+ vs. MetS−			
	SAT		VAT	
Inflammation	FC	*p*-value	FC	*p*-value
*CD68*	1.47	0.019	1.27	0.33
*CD80* *	3.00	0.004	2.00	0.125
*MRC1*	1.35	0.03	1.11	0.338
*MSR1*	1.49	0.131	1.28	0.045
*HIF1A*	1.52	0.024	0.94	0.675
Adipokines				
*ADIPOQ* *	1.39	0.042	0.94	0.315
*LEPR* *	0.62	0.009	1.23	0.436
*FABP4*	1.22	0.295	0.53	0.003
Angiogenesis				
*VEGFA* *	1.21	0.115	0.96	0.826
Glucose metabolism				
*GLUT1*	1.00	0.085	1.33	0.044
Lipogenesis				
*FASN*	0.53	0.012	0.64	0.009
*MOGAT1*	1.50	0.028	2.00	0.313
FAO/Beiging				
*PPARA* *	1.25	0.008	1.03	0.825
*UCP1*	1.00	0.603	0.40	0.017
*ADRB1*	1.73	0.028	1.12	0.477
*ADRB3*	1.00	0.842	0.33	0.004
*PRDM16*	2.00	0.031	1.00	0.434
*CIDEA*	1.13	0.406	0.65	0.002
*CPT1A* *	1.37	0.006	1.41	0.003
ECM remodelling				
*TGFB1*	1.34	0.033	1.32	0.01
*F13A1*	1.71	0.026	1.61	0.028
*COL5A1*	1.36	0.168	0.68	0.036
*MMP2*	1.09	0.74	0.51	0.016
*MMP13*	1.00	0.882	0.10	0.0005
*TIMP1*	1.70	0.331	0.45	0.002
*HYAL1*	1.00	0.185	0.30	<0.0001
*HYAL2*	1.36	0.603	0.29	0.001
*LOXL2*	1.48	0.025	1.02	0.91
*LOXL4*	1.44	0.044	2.75	0.194

MetS−, severely obese without MetS; MetS+, severely obese with MetS; SAT, subcutaneous adipose tissue; VAT, visceral adipose tissue; FC, fold change; FAO, fatty acid oxidation; ECM extracellular matrix. * Genes included in the PLS-DA model for the identification of MetS.

**Table 4:** Regression analysis model of the adipose tissue gene expression associated to MetS.

Genes	B	S.E. (B)	Exp B (OR) (95% CI)	Sig.	R2 (%)	Correct Prediction (%)	M. Sig.
Component 1							
SAT-LEPR	−15.798	6.450	0.12 [0.04—0.43]	0.014	64.4	85.2	0.000
SAT-PPARA	17.9	7.365	6.01 [1.27—12.95]	0.015			
Constant	−3.509	2.649	0.30	0.185			

B, beta coefficient; S.E. (B), standard error of beta: Exp B (OR (95% CI); exponent beta with 95% confidence intervals; Sig, *p*-value tested for each independent variable enter in the model; R2, Nagelkerke R square of the model; Correct prediction (%), percentage of patients correctly classified for NAFLD according to model; M. Sig; model significance based on Omnibus test of the model coefficients.

## Data Availability

All data presented in this study are reported in this manuscript or available in the Supplementary Materials.

## Supplementary Materials

The following supporting information can be downloaded at https://www.mdpi.com/article/10.3390/ijms23042394/s1.
