# Supplementary material for: Biological Determinants of Metabolic Syndrome in Visceral and Subcutaneous Adipose Tissue from Severely Obese Women
Source: Int J Mol Sci. 2022 Feb 21;23(4):2394. doi: 10.3390/ijms23042394 (PMC8878297; doi:10.3390/ijms23042394)
Supplement: Supplementary file 1 [file ijms-23-02394-s001.zip › ijms-1566292-supplementary.pdf]

Supplementary Table S1

	MetS- (n=33)				MetS+ (n=33)				MetS+ vs MetS-			
	SAT	VAT	FC	P-value	SAT	VAT	FC	P-value	SAT		VAT	
**Inflammation**									FC	P-value	FC	P-value
CD68	0.849 ± 0.425	0.674 ± 0.175	0.79	0.118	1.246 ± 0.769	0.853 ± 0.434	0.68	**0.022***	1.47	**0.019#**	1.27	0.33
CD80	0.001 ± 0.001	0.001 ± 0.001	1.00	0.515	0.003 ± 0.001	0.002 ± 0.001	0.67	0.071	3.00	**0.004#**	2.00	0.125
CD14	0.423 ± 0.225	0.412 ± 0.21	0.97	0.895	0.598 ± 0.288	0.506 ± 0.234	0.85	0.268	1.41	0.074	1.23	0.161
MRC1	0.113 ± 0.072	0.143 ± 0.061	1.27	**0.016***	0.153 ± 0.087	0.159 ± 0.064	1.04	0.58	1.35	**0.03#**	1.11	0.338
MSR1	0.156 ± 0.107	0.1 ± 0.036	0.64	0.061	0.233 ± 0.152	0.128 ± 0.056	0.55	**0.009***	1.49	0.131	1.28	**0.045#**
MCP1	0.502 ± 0.49	0.953 ± 0.936	1.90	0.078	0.842 ± 1.5	0.896 ± 1.033	1.06	0.178	1.68	0.805	0.94	0.583
HIF1A	0.23 ± 0.08	0.331 ± 0.156	1.44	**0.026***	0.349 ± 0.196	0.312 ± 0.133	0.89	0.4502	1.52	**0.024#**	0.94	0.675
IL1B	0.019 ± 0.019	0.162 ± 0.273	8.53	**0.013***	0.026 ± 0.039	0.185 ± 0.287	7.12	**0.011***	1.37	0.869	1.14	0.869
IL6	0.293 ± 0.385	0.408 ± 0.548	1.39	0.489	0.451 ± 0.889	0.301 ± 0.363	0.67	0.568	1.54	0.567	0.74	0.609
TNFA	0.0002 ± 0.0002	0.001 ± 0.002	5.00	**0.008***	0.001 ± 0.001	0.002 ± 0.005	2.00	**0.036***	5.00	0.353	2.00	0.625
PAI-1	0.074 ± 0.049	0.192 ± 0.145	2.59	**0.014***	0.16 ± 0.16	0.207 ± 0.245	1.29	0.388	2.16	0.069	1.08	0.749
**Adipokines**												
ADIPOQ	4.35 ± 1.966	3.592 ± 1.093	0.83	0.074	5.43 ± 2.802	3.359 ± 1.686	0.62	**0.001***	1.39	0.042	0.94	0.315
LEP	1.217 ± 0.943	0.419 ± 0.344	0.34	**<0.0001***	1.165 ± 0.826	0.44 ± 0.276	0.38	**0.0002***	0.96	0.986	1.05	0.481
ADIPOR1	0.204 ± 0.075	0.196 ± 0.083	0.96	0.608	0.198 ± 0.061	0.163 ± 0.044	0.82	**0.039***	0.97	0.783	0.83	0.267
ADIPOR2	0.189 ± 0.08	0.181 ± 0.077	0.96	0.887	0.201 ± 0.095	0.163 ± 0.072	0.81	0.149	1.06	0.55	0.90	0.466
LEPR	0.333 ± 0.175	0.27 ± 0.024	0.81	0.697	0.241 ± 0.123	0.331 ± 0.094	1.37	**0.027***	0.62	0.009	1.23	0.436
NPY1R	0.112 ± 0.058	0.067 ± 0.029	0.60	**0.021***	0.138 ± 0.074	0.066 ± 0.03	0.48	**0.002***	1.23	0.347	0.99	0.619
**Senescence**												
p16	0.005 ± 0.004	0.004 ± 0.004	0.80	0.335	0.006 ± 0.008	0.003 ± 0.005	0.50	0.215	1.20	0.548	0.75	0.601
p21	0.058 ± 0.063	0.152 ± 0.106	2.62	**0.006***	0.072 ± 0.068	0.139 ± 0.188	1.93	0.265	1.24	0.928	0.91	0.203
P53	0.069 ± 0.031	0.055 ± 0.018	0.80	0.166	0.086 ± 0.044	0.055 ± 0.017	0.64	**0.008***	1.25	0.24	1.00	0.885
**Autophagy**												
ATG5	0.062 ± 0.012	0.057 ± 0.009	0.92	0.202	0.06 ± 0.017	0.059 ± 0.014	0.98	0.884	0.97	0.829	1.04	0.416
ATG7	0.038 ± 0.011	0.046 ± 0.011	1.21	0.064	0.042 ± 0.013	0.041 ± 0.01	0.98	0.85	1.11	0.437	0.89	0.144
ATG12	0.071 ± 0.019	0.067 ± 0.013	0.94	0.480	0.085 ± 0.033	0.068 ± 0.015	0.80	0.129	1.20	0.231	1.01	0.931
**Adipogenesis**												
PPARG	0.514 ± 0.116	0.419 ± 0.176	0.82	0.108	0.486 ± 0.175	0.341 ± 0.15	0.70	**0.021***	0.95	0.595	0.81	0.234
PPARA	0.038 ± 0.011	0.033 ± 0.008	0.87	0.129	0.049 ± 0.011	0.034 ± 0.008	0.69	**<0.0001***	1.29	**0.001#**	1.03	0.825
FABP4	6.089 ± 1.677	4.481 ± 1.637	0.74	**0.02***	7.432 ± 4.142	2.392 ± 1.851	0.32	**0.002***	1.22	0.295	0.53	**0.003#**
PDGFRA	0.108 ± 0.052	0.134 ± 0.044	1.24	**0.021***	0.115 ± 0.043	0.129 ± 0.052	1.12	0.294	1.06	0.303	0.96	0.655
PDGFRB	0.281 ± 0.104	0.156 ± 0.06	0.56	**0.001***	0.282 ± 0.109	0.148 ± 0.059	0.52	**<0.0001***	1.00	0.972	0.95	0.712
BMP2	0.024 ± 0.012	0.032 ± 0.016	1.33	0.123	0.022 ± 0.01	0.026 ± 0.014	1.18	>0.9999	0.92	0.685	0.81	0.316
BMP4	0.032 ± 0.018	0.038 ± 0.023	1.19	0.6139	0.037 ± 0.016	0.04 ± 0.019	1.08	0.626	1.16	0.392	1.05	0.418
**Angiogenesis**												
VEGFA	0.036 ± 0.015	0.051 ± 0.027	1.42	**0.016***	0.04 ± 0.017	0.049 ± 0.021	1.23	0.103	1.21	0.115	0.96	0.826
VEGFB	0.259 ± 0.08	0.233 ± 0.076	0.90	0.159	0.323 ± 0.14	0.254 ± 0.1	0.79	0.062	1.25	0.152	1.09	0.596
VEGFR1	0.159 ± 0.057	0.179 ± 0.078	1.13	0.319	0.162 ± 0.07	0.184 ± 0.107	1.14	0.839	1.02	0.88	1.03	0.826
VEGFR2	0.106 ± 0.045	0.12 ± 0.045	1.13	0.593	0.105 ± 0.041	0.125 ± 0.048	1.19	0.135	0.99	0.967	1.04	0.321
ANGPT1	0.031 ± 0.014	0.024 ± 0.01	0.77	0.140	0.038 ± 0.024	0.028 ± 0.014	0.74	0.052	1.23	0.175	1.17	0.53
ANGPT2	0.03 ± 0.016	0.023 ± 0.01	0.77	0.195	0.041 ± 0.033	0.027 ± 0.015	0.66	**0.044***	1.37	0.22	1.17	0.749

Supplementary Table S1. Expression analysis of inflammation markers, adipokines and senescence, autophagy, adipogenesis and angiogenesis-related genes. MetS-, severely obese without MetS; MetS+, severely obese with MetS; SAT, subcutaneous adipose tissue; VAT, visceral adipose tissue. Data expressed as mean ± SD. *= P<0.05 between depots from the same group. #= P<0.05 respect to the same depot from the other group.

Supplementary Table S2

	MetS- (n=33)				MetS+ (n=33)				MetS+ vs MetS-			
	SAT	VAT	FC	P-value	SAT	VAT	FC	P-value	SAT		VAT	
**Glucose metabolism**									FC	P-value	FC	P-value
GLUT1	0.003 ± 0.001	0.003 ± 0.001	1.00	0.49	0.003 ± 0.002	0.004 ± 0.003	1.33	0.367	1.00	0.085	1.33	**0.044**#
GLUT4	0.05 ± 0.025	0.048 ± 0.03	0.96	0.683	0.058 ± 0.024	0.045 ± 0.026	0.78	0.211	1.16	0.473	0.94	0.683
IRS1	0.024 ± 0.013	0.013 ± 0.008	0.54	**0.005***	0.024 ± 0.012	0.013 ± 0.008	0.54	**0.01***	1.00	0.9	1.00	0.994
**Lipolysis**												
KLB	0.166 ± 0.108	0.708 ± 1.706	4.27	0.785	0.214 ± 0.158	1.589 ± 3.976	7.43	0.3175	1.29	0.388	2.24	0.545
PLIN1	2.527 ± 0.751	2.012 ± 0.568	0.80	0.061	2.757 ± 1.299	1.768 ± 0.861	0.64	**0.045***	1.09	0.592	0.88	0.401
PLIN2	0.593 ± 0.428	0.199 ± 0.094	0.34	**0.0002***	0.439 ± 0.213	0.233 ± 0.077	0.53	**0.0007***	0.74	0.563	1.17	0.092
ATGL	2.784 ± 0.886	2.269 ± 1.137	0.82	0.055	3.297 ± 1.778	2.466 ± 1.164	0.75	0.218	1.18	0.369	1.09	0.615
HSL	0.149 ± 0.188	0.076 ± 0.13	0.51	0.212	0.224 ± 0.406	0.168 ± 0.172	0.75	0.943	1.50	0.676	2.21	0.488
MGLL	1.335 ± 0.575	0.465 ± 0.131	0.35	**0.013***	1.584 ± 0.837	0.5 ± 0.14	0.32	**0.002***	1.19	0.532	1.08	0.587
**Lipogenesis**												
SREBF1	0.09 ± 0.031	0.051 ± 0.015	0.57	**<0.0001***	0.108 ± 0.076	0.046 ± 0.04	0.43	**0.005***	1.20	0.989	0.90	0.687
FASN	0.521 ± 0.407	0.406 ± 0.219	0.78	0.618	0.278 ± 0.184	0.26 ± 0.123	0.94	0.713	0.53	**0.012**#	0.64	**0.009**#
MOGAT1	0.002 ± 0.001	0.001 ± 0.001	0.50	0.12	0.003 ± 0.002	0.002 ± 0.001	0.67	0.069	1.50	**0.028**#	2.00	0.313
DGAT2	0.757 ± 0.372	0.306 ± 0.235	0.40	**<0.0001***	0.872 ± 0.403	0.386 ± 0.314	0.44	**0.0008***	1.15	0.475	1.26	0.276
LPL	2.884 ± 1.201	2.27 ± 1.147	0.79	0.095	3.14 ± 1.515	2.251 ± 1.039	0.72	**0.036***	1.09	0.527	0.99	0.873
ABCA1	0.223 ± 0.065	0.172 ± 0.046	0.77	**0.03***	0.263 ± 0.104	0.158 ± 0.084	0.60	**0.005***	1.18	0.27	0.92	0.186
APOE	0.014 ± 0.008	0.003 ± 0.002	0.21	**<0.0001***	0.011 ± 0.007	0.003 ± 0.002	0.27	**<0.0001***	0.79	0.145	1.00	0.453
**Beiging / FAO**												
UCP1	0.001 ± 0.002	0.005 ± 0.005	5.00	**0.0005***	0.001 ± 0.001	0.002 ± 0.002	2.00	0.055	1.00	0.603	0.40	**0.017**#
UCP2	0.654 ± 0.102	0.4 ± 0.152	0.61	0.451	0.607 ± 0.334	0.437 ± 0.179	0.72	0.095	0.93	0.18	1.09	0.614
UCP3	0.001 ± 0.001	0.001 ± 0.0004	1.00	0.118	0.002 ± 0.001	0.001 ± 0.0004	0.50	0.054	2.00	0.323	1.00	0.964
DIO2	0.008 ± 0.003	0.003 ± 0.002	0.38	**0.002***	0.009 ± 0.006	0.003 ± 0.001	0.33	**0.044***	1.13	0.642	1.00	0.776
PGC1A	0.006 ± 0.003	0.008 ± 0.003	1.33	**0.002***	0.005 ± 0.003	0.007 ± 0.003	1.40	**0.036***	0.83	0.515	0.88	0.426
ADRB1	0.011 ± 0.005	0.025 ± 0.014	2.27	**0.0001***	0.019 ± 0.009	0.028 ± 0.013	1.47	0.058	1.73	**0.028**#	1.12	0.477
ADRB3	0.001 ± 0.001	0.003 ± 0.003	3.00	0.146	0.001 ± 0.001	0.001 ± 0.0008	1.00	0.151	1.00	0.842	0.33	**0.004**#
PRDM16	0.001 ± 0.0005	0.003 ± 0.0008	3.00	**0.009***	0.002 ± 0.0006	0.003 ± 0.001	1.50	**0.005***	2.00	**0.031**#	1.00	0.434
PGC1B	0.005 ± 0.002	0.009 ± 0.002	1.80	**0.002***	0.006 ± 0.002	0.008 ± 0.003	1.33	0.09	1.20	0.347	0.89	0.813
CIDEA	0.015 ± 0.005	0.034 ± 0.009	2.27	**0.0007***	0.017 ± 0.008	0.022 ± 0.008	1.29	0.169	1.13	0.406	0.65	**0.002**#
CPT1A	0.046 ± 0.012	0.034 ± 0.008	0.74	**0.016***	0.062 ± 0.014	0.021 ± 0.007	0.34	**<0.0001***	1.37	**0.006**#	1.41	**0.003**#
ACOX1	0.297 ± 0.118	0.178 ± 0.11	0.60	**0.005***	0.268 ± 0.118	0.216 ± 0.092	0.81	0.195	0.90	0.547	1.21	0.081

Supplementary Table S2. Expression analysis of genes involved in glucose and lipid metabolism, fatty acid oxidation and beiging. MetS-, severely obese without MetS; MetS+, severely obese with MetS; SAT, subcutaneous adipose tissue; VAT, visceral adipose tissue; FAO, fatty acid oxidation. Data expressed as mean ± SD. *= P<0.05 between depots from the same group. #= P<0.05 respect to the same depot from the other group.

Supplementary Table S3

	MetS- (n=33)				MetS+ (n=33)				MetS+ vs MetS-			
	SAT	VAT	FC	P-value	SAT	VAT	FC	P-value	SAT		VAT	
**ECM remodeling**									FC	P-value	FC	P-value
TCF21	0.0004 ± 0.0002	0.011 ± 0.004	27.50	**<0.0001***	0.0004 ± 0.0003	0.011 ± 0.001	27.50	**<0.0001***	1.00	>0.9999	1.00	0.978
TGFB1	0.134 ± 0.055	0.098 ± 0.03	0.73	**0.003***	0.18 ± 0.099	0.129 ± 0.051	0.72	**0.04***	1.34	**0.033#**	1.32	**0.01#**
F13A1	0.335 ± 0.196	0.287 ± 0.135	0.86	0.506	0.572 ± 0.392	0.461 ± 0.296	0.81	0.427	1.71	**0.026#**	1.61	**0.028#**
COL1A1	0.512 ± 0.445	2.562 ± 3.102	5.00	**0.014***	0.516 ± 0.313	4.927 ± 7.962	9.55	**0.041***	1.01	0.717	1.92	0.920
COL3A1	0.168 ± 0.11	0.474 ± 0.237	2.82	**0.0004***	0.269 ± 0.243	0.403 ± 0.135	1.50	**0.039***	1.60	0.266	0.85	0.393
COL4A1	1.711 ± 0.699	4.596 ± 7.885	2.69	>0.9999	2.275 ± 1.188	8.014 ± 14.524	3.52	0.373	1.33	0.172	1.74	0.758
COL5A1	0.177 ± 0.053	0.326 ± 0.146	1.84	**0.0087***	0.241 ± 0.123	0.222 ± 0.050	0.92	0.656	1.36	0.168	0.68	**0.036#**
COL6A1	0.02 ± 0.016	0.022 ± 0.034	1.10	0.156	0.044 ± 0.066	0.048 ± 0.07	1.09	0.334	2.20	0.228	2.18	0.265
COL6A3	0.353 ± 0.148	0.182 ± 0.135	0.52	**<0.0001***	0.411 ± 0.188	0.189 ± 0.076	0.46	**<0.0001***	1.16	0.386	1.04	0.373
MMP2	0.685 ± 0.238	1.495 ± 0.889	2.18	**0.015***	0.744 ± 0.474	0.766 ± 0.288	1.03	0.641	1.09	0.74	0.51	**0.016#**
MMP9	0.211 ± 0.217	0.039 ± 0.034	0.18	**<0.0001***	0.288 ± 0.325	0.038 ± 0.025	0.13	**<0.0001***	1.36	0.387	0.97	0.712
MMP13	0.002 ± 0.002	0.001 ± 0.001	0.50	0.887	0.002 ± 0.003	0.0001 ± 0.0002	0.05	**0.0006***	1.00	0.882	0.10	**0.0005#**
MMP14	0.161 ± 0.076	0.431 ± 0.165	2.68	**<0.0001***	0.198 ± 0.11	0.401 ± 0.113	2.03	**0.0004***	1.23	0.401	0.93	0.804
MMP15	0.021 ± 0.01	0.045 ± 0.054	2.14	0.773	0.029 ± 0.022	0.026 ± 0.017	0.90	0.945	1.38	0.402	0.58	0.954
TIMP1	0.091 ± 0.037	1.330 ± 0.654	14.62	**<0.0001***	0.155 ± 0.157	0.603 ± 0.268	3.89	**0.0002***	1.70	0.331	0.45	**0.002#**
TIMP2	0.481 ± 0.224	0.359 ± 0.059	0.75	**0.03***	0.66 ± 0.523	0.343 ± 0.049	0.52	0.175	1.37	0.521	0.96	0.652
TIMP3	0.824 ± 0.39	3.247 ± 1.769	3.94	**0.0007***	0.979 ± 0.67	2.731 ± 1.464	2.79	**0.0008***	1.19	0.882	0.84	0.761
HYAL1	0.023 ± 0.029	0.221 ± 0.141	9.61	**<0.0001***	0.023 ± 0.014	0.066 ± 0.029	2.87	**< 0.0001***	1.00	0.185	0.30	**< 0.0001#**
HYAL2	0.247 ± 0.093	1.774 ± 1.095	7.18	**<0.0001***	0.335 ± 0.269	0.506 ± 0.297	1.51	0.0648	1.36	0.603	0.29	**0.001#**
LOX	0.206 ± 0.091	0.625 ± 0.349	3.03	**0.002***	0.254 ± 0.159	0.441 ± 0.117	1.74	**0.005***	1.23	0.44	0.71	0.111
BGN	0.287 ± 0.151	0.192 ± 0.069	0.67	**0.005***	0.392 ± 0.408	0.185 ± 0.036	0.47	**0.003***	1.37	0.387	0.96	0.776
LOXL2	0.12 ± 0.057	0.052 ± 0.027	0.43	**<0.0001***	0.177 ± 0.106	0.053 ± 0.019	0.30	**<0.0001***	1.48	**0.025#**	1.02	0.91
LOXL4	0.009 ± 0.004	0.008 ± 0.009	0.89	**0.028***	0.013 ± 0.01	0.022 ± 0.031	1.69	0.396	1.44	**0.044#**	2.75	0.194
HAS1	0.006 ± 0.008	0.813 ± 1.343	135.50	**<0.0001***	0.009 ± 0.008	2.316 ± 6.443	257.33	**<0.0001***	1.50	0.056	2.85	0.928
HAS2	0.01 ± 0.004	0.016 ± 0.017	1.60	0.702	0.02 ± 0.019	0.01 ± 0.005	0.50	0.287	2.00	0.503	0.63	0.629
CD44	0.007 ± 0.004	0.003 ± 0.002	0.43	**0.001***	0.011 ± 0.01	0.004 ± 0.006	0.36	**0.004***	1.57	0.656	1.33	0.525
ELN	0.203 ± 0.08	0.297 ± 0.171	1.46	0.097	0.327 ± 0.286	0.221 ± 0.086	0.68	0.56	1.61	0.403	0.74	0.196
FN1	0.49 ± 0.255	0.365 ± 0.329	0.74	0.094	0.749 ± 0.938	0.43 ± 0.336	0.57	0.442	1.53	0.882	1.18	0.460

Supplementary Table S3. Expression analysis of genes involved in ECM composition and remodeling. MetS-, severely obese without MetS; MetS+, severely obese with MetS; SAT, subcutaneous adipose tissue; VAT, visceral adipose tissue; ECM, extracellular matrix. Data expressed as mean ± SD. *= P<0.05 between depots from the same group. #= P<0.05 respect to the same depot from the other group.

Supplementary Table S4

GENE	SEQUENCE	(5'-3')
ABCA1	F	GGAGGCCAGAATGACATCTTAG
ABCA1	R	TTTCCAGCCCCATTAACTCC
ACOX1	F	ACCATTGCCATCCGATACAG
ACOX1	R	GGTCTCCTTCATGTATGCGC
ADFP/PLIN2	F	AGTATCCCTACCTGAAGTCTGTG
ADFP/PLIN2	R	CCCCTTACAGGCATAGGTATTG
ADIPOQ	F	ACAATGACTCCACCTTCACAG
ADIPOQ	R	TTCCTAACCGTACTGAAAGCC
ADRB1	F	CCGGGAACAGGAACACAC
ADRB1	R	GAAAGCAAAAGGAAATATGTCTTGA
ADRB3	F	TTTTCTAAACCCCAGCCTTG
ADRB3	R	CACGGCACCTGGACACTAC
ANGPT1	F	TTAAAGGACTTACAGGGACAGC
ANGPT1	R	GACCACATGCATCAAACCAC
ANGPT2	F	CCACGAGACTTGAACTTCAGC
ANGPT2	R	TGTGCTTGTCTTCCATAGCTAG
APOE	F	CAGCGACAATCACTGAACG
APOE	R	GTGAATCTTTATTAAACTAGGGTCCAC
ATG12	F	AATCAGTCCTTTGCTCCTTCC
ATG12	R	GCAAGTTGATTTTCTTTGTGGTTC
ATG5	F	AGCAACTCTGGATGGGATTG
ATG5	R	AGGTCTTTCAGTCGTTGTCTG
ATG7	F	TTTTGCTATCCTGCCCTCTG
ATG7	R	GCTGTGACTCCTTCTGTTTGAC
ATGL	F	CACTTCAACTCCAAGGACGAG
ATGL	R	CTCATAGAGTGGCAGGTTGTC
BMP2	F	CTATCAGGACATGGTTGTGGAG
BMP2	R	GGGAAATATTAAAGTGTCAACTGGG
BMP4	F	TGGCTGTCAAGAATCATGGAC
BMP4	R	CCCGTCTCAGGTATCAAACTAG
CD14	F	CAGAGGTTCGGAAGACTTATCG
CD14	R	TTCGGAGAAGTTGCAGACG
CD206/MRC1	F	GCAAAGTGGATTACGTGTCTTG
CD206/MRC1	R	CTGTTATGTCGCTGGCAAATG
CD68	F	ATGGCGGTGGAGTACAATG
CD68	R	TGGACAGCTGGTGAAAGAATG
CD80	F	CCATCCAAGTGTCCATACCTC
CD80	F	CTCACTTCTGTTCAGGTGTTATCCA
CD80	R	GCCAGCTCTTCAACAGAAAC
CD80	R	TCCTTTTGCCAGTAGATGCGA
CD86	F	ACATTCTCTTTGTGATGGCCTTC
CD86	R	TGCAGTCTCATTGAAATAAGCTTGA
CIDEA	F	GGCAGGTTCACGTGTGGATA
CIDEA	R	GAAACACAGTGTTTGGCTCAAGA
CPT1A	F	TCCAGTTGGCTTATCGTGGTG
CPT1A	R	CTAACGAGGGGTCGATCTTGG
DGAT2	F	TCCGAATGCCTGTGTTGAG
DGAT2	R	CAAATAGTCTATGGTGTCCCGG
FABP4	F	CATGTGCAGAAATGGGATGG
FABP4	R	AACTTCAGTCCAGGTCAACG
FASN	F	CAGAGTCGGAGAACTTGCAG
FASN	R	GGAGGCATCAAACCTAGACAG
HIF1A	F	AAGAACTTTTAGGCCGCTCA
HIF1A	R	CAACCCAGACATATCCACCTC
HSL/LIPE	F	TCATCTCCATCGACTACTCCC
HSL/LIPE	R	AGATTCGTTCCCCTGTTGAG
IL6	F	CAACCTGAACCTTCCAAAGATG
IL6	R	ACCTCAAACTCCAAAAGACCAG
IRS1	F	TCTGCTCAGCGTTGGTG
IRS1	R	GTGCATGCTCTTGGGTTTG
KLB	F	CATGGGTATGGGACAGGTATG
KLB	R	TCTGATGTGGGCGGAAATG
LEP	F	GCTTCAGGCTACTCCACAG
LEP	R	CCTTCCCTTAACGTAGTCCTTG
LEPR	F	TCAACCAGTACAATCCAGTCAC
LEPR	R	TTTGGGCTCAGATATGGGATG
LPL	F	GGACTGAGAGTGAAACCCATAC
LPL	R	GGAAGGAGTAGGTCTTATTTGTGG
MCP1/CCL2	F	CCTCCAGCATGAAAGTCTCTG
MCP1/CCL2	R	TCTGCACTGAGATCTTCCTATTG

GENE	SEQUENCE	(5'-3')
MGLL	F	AGCATGCCAGAGGAAAGTTC
MGLL	R	ATGGGACACAAAGATGAGGG
MOGAT1	F	GAAAGCCATCCACACTGTTG
MOGAT1	R	GCCATACTTTCCTTTGTGTTCC
MSR1	F	ATCTGTGAAATTTGATGCTCGC
MSR1	R	CCAATGAGAGGGATGAGAACTG
P16/CDKN2A	F	GATGTCGCACGGTACCTG
P16/CDKN2A	R	TCTCTGGTTCTTTCAATCGGG
P21/CDKN1A	F	GAACTTCGACTTTGTCACCGAGAC
P21/CDKN1A	R	TGGAGTGGTAGAAATCTGTCATGCT
P53/TP53	F	CAGCACATGACGGAGGTTGT
P53/TP53	R	TCATCCAAATACTCCACACGC
PAI-1/SERPINE1	F	GTGGACTTTTCAGAGGTGGAG
PAI-1/SERPINE1	R	GAAGTAGAGGGCATTCACCAG
PDGFRA	F	TTCCTCTGCCTGACATTGAC
PDGFRA	R	GTCTTCAATGGTCTCGTCCTC
PDGFRB	F	ATGTGACGGAGAGTGTGAATG
PDGFRB	R	GCAGCTCAGCAAATTGTAGTG
PGC1B	F	GTACATTCAAAATCTCTCCAGCGACAT
PGC1B	R	GAGGGCTCGTTGCGCTTCCTCAGGGC
PLIN1	F	CATTGAGAAGGTGGTGGAGTAC
PLIN1	R	GTGTATCGAGAGAGGGTGTTG
PLIN2	F	AGTATCCCTACCTGAAGTCTGTG
PLIN2	R	CCCCTTACAGGCATAGGTATTG
PPARA	F	CTATCATTTGCTGTGGAGATCG
PPARA	R	AAGATATCGTCCGGGTGGTT
PPARG	F	GTCGGTTTCAGAAATGCCTTG
PPARG	R	GCTGGTCGATATCACTGGAG
PPARGC1A/PGC1A	F	CAGGCAGTAGATCCTCTTCAAG
PPARGC1A/PGC1A	R	TCCTCGTAGCTGTCATACCTG
PRDM16	F	CACGAGTGCAAGGACTGC
PRDM16	R	TGTGGATGACCATGTGCTG
RPL6	F	CCTTAATTCTCTTTCCCATCTTGC
RPL6	R	TTCTTGGCTTCGGGTTTCTT
SDHA	F	TGGTTGTCTTTGGTCGGG
SDHA	R	GCGTTTGGTTTAATTGGAGGG
SLC2A1/GLUT1	F	TCATCGTGGCTGAACTCTTC
SLC2A1/GLUT1	R	GATGAAGACGTAGGGACCAC
SLC2A4/GLUT4	F	ACTGGACGAGCAACTTCATC
SLC2A4/GLUT4	R	GAGGACCGCAAATAGAAGGAA
SOD2	F	GACAAACCTCAGCCCTAACG
SOD2	R	GAAACCAAGCCAACCCCAAC
SREBF1	F	TTCTGACAGCCATGAAGACAG
SREBF1	R	CCGCATCTACGACCAGTG
TGFB1	F	TTGATGTCACCGGAGTTGTG
TGFB1	R	GTAGTGAACCCGTTGATGTCC
TNFA	F	AGGTCTACTTTGGGATCATTGC
TNFA	R	GAAGAGGTTGAGGGTGTCTG
UCP1	F	GGACTACTCCCAATCTGATGAG
UCP1	R	AAATCCAGCGATAAGAGCCG
UCP2	F	TCCTGAAAGCCAACCTCATG
UCP2	R	GGCAGAGTTCATGTATCTCGTC
UCP3	F	AGAAAATACAGCGGGACTATGG
UCP3	R	CTTGAGGATGTCGTAGGTCAC
VEGFA	F	AGTCCAACATCACCATGCAG
VEGFA	R	TTCCCTTTCCTCGAACTGATTT
VEGFB	F	CTTAGAGCTCAACCCAGACAC
VEGFB	R	ACCCTGCTGAGTCTGAAAAG
VEGFR1	F	TCCCTCAACCTACAATCAAGTG
VEGFR1	R	GCTCTCAATTCTGTTTCCCATG
VEGFR2	F	CATTTCAAAGGAGAAGCAGAGC
VEGFR2	R	GAGGAATGGCATAGACCGTAC

Supplementary Table S4. List of oligonucleotides. F, forward; R, reverse.

Supplementary Figure S1

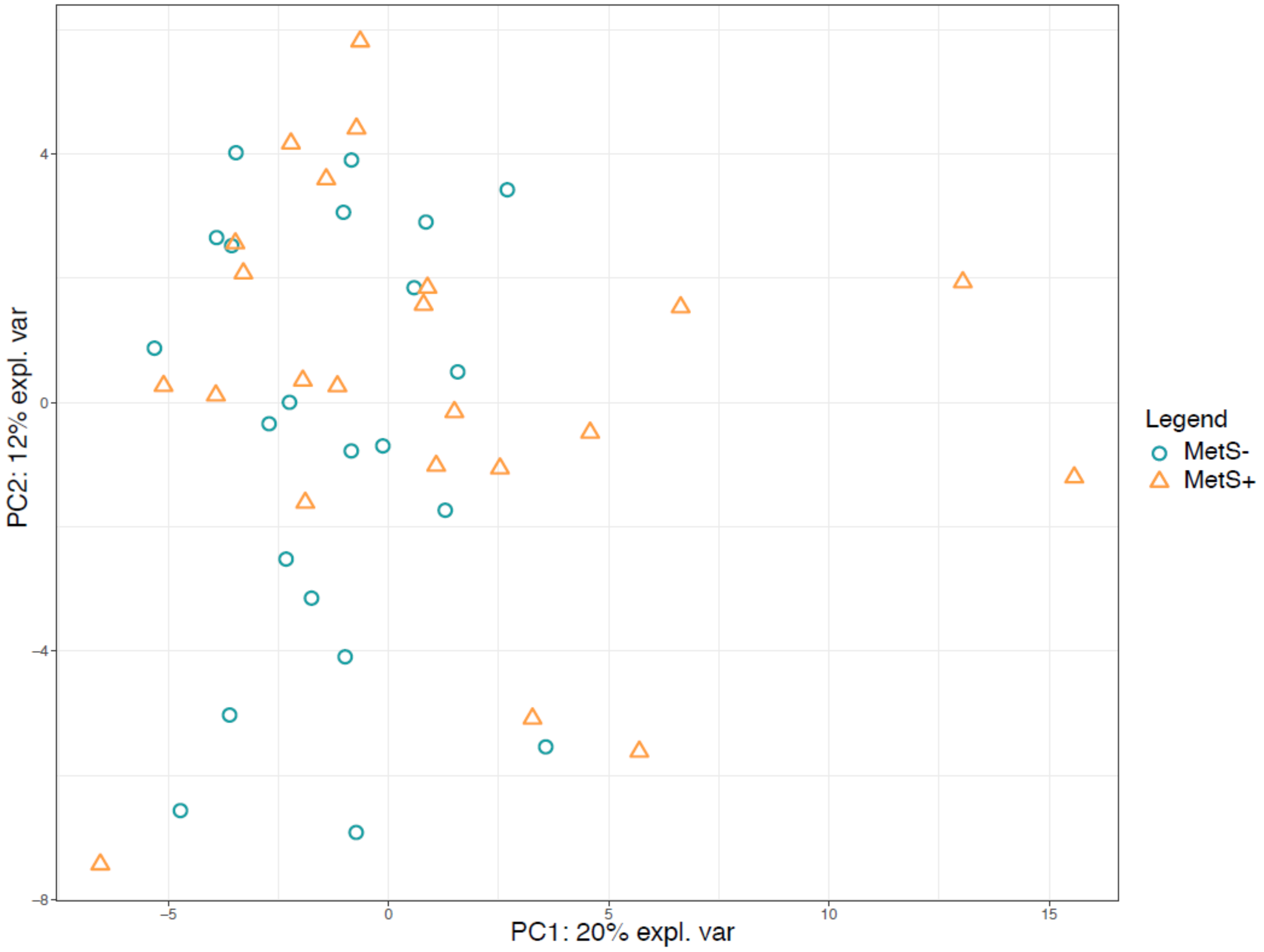

Supplementary Figure S1. Unsupervised PCA including expression data from all 93 genes.
